# Temporal Coherence of Single Photons Emitted by Hexagonal Boron Nitride Defects at Room Temperature

**DOI:** 10.1021/acsphotonics.5c02227

**Published:** 2025-12-27

**Authors:** Juan Vidal Martínez-Pons, Sang Kyu Kim, Max Behrens, Alejandro Izquierdo-Molina, Adolfo Menendez Rua, Serkan Paçal, Serkan Ateş, Luis Viña, Carlos Antón-Solanas

**Affiliations:** † Depto. de Física de Materiales, 16722 Universidad Autónoma de Madrid, 28049 Madrid, Spain; ‡ Instituto Nicolás Cabrera, 9184 Universidad Autónoma de Madrid, 28049 Madrid, Spain; § Walter Schottky Institut, Institute for Advanced Study, TUM School of Computation, Information and Technology, and MCQST, Technische Universität München, 85748 Garching, Germany; ∥ Department of Physics, Izmir Institute of Technology, Izmir 35430, Turkey; ⊥ Faculty of Engineering and Natural Sciences, 52991 Sabanci University, 34956 Tuzla, Istanbul Turkey; a Centro de Física de la Materia Condensada (IFIMAC), 52972 Universidad Autónoma de Madrid, 28049 Madrid, Spain

**Keywords:** hBN defects, single-photon emitters, quantum optics, temporal coherence, phonon dephasing, michelson interferometry

## Abstract

Color centers in hexagonal boron nitride (hBN) emerge as promising quantum light sources at room temperature, with potential applications in quantum communications, among others. The temporal coherence of emitted photons (i.e., their capacity to interfere and distribute photonic entanglement) is essential for many of these applications. Hence, it is crucial to study and determine the temporal coherence of this emission under different experimental conditions. In this work, we report the coherence time of the single photons emitted by an hBN defect in a nanocrystal at room temperature, measured via Michelson interferometry. The visibility of this interference vanishes when the temporal delay between the interferometer arms is a few hundred femtoseconds, highlighting that the phonon dephasing processes are 4 orders of magnitude faster than the spontaneous decay time of the emitter. We also analyze the single photon characteristics of the emission via correlation measurements, defect blinking dynamics, and its Debye–Waller factor. Our room temperature results highlight the presence of a strong electron–phonon coupling, suggesting the need to work at cryogenic temperatures to enable quantum photonic applications based on photon interference.

## Graphical abstract

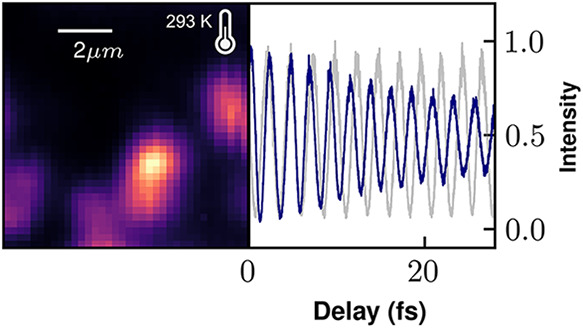

## Introduction

Quantum optical technologies, such as communication, computation or metrology, demand the development of optimal quantum light sources. The two crucial characteristics of an optimal single photon source are its efficiency to generate a single photon per excitation drive, and its temporal coherence, determining its capacity to interfere and distribute entanglement.^1^ Considering solid-state sources, the state-of-the-art performance is achieved by self-assembled semiconductor quantum dots (QDs) weakly coupled to optical cavities.^2,3^ These results have shown record source-to-detector efficiency of *B*_d_ > 55%; in natural atoms coupled to cavities, this value is <45%.^4^ Prominent solid-state emitters, among many others,^5,6^ are nitrogen- and silicon-vacancy centers,^7,8^ demonstrating fundamental applications in sensing and communications, and organic molecules,^9^ which present a promising route to implement multiemitter systems via engineered dipole coupling.^10,11^ Over the past decade, other solid-state emitters have gained relevance, such as QDs in monolayers of transition metal dichalcogenides,^12–16^ and defects in hBN.^17^ Our studies along this work are based on such single photon emitter.

Two key parameters of single photon emission performance are the intrinsic quantum efficiency of the source (ratio of the radiative spontaneous decay rate to the total decay rate) and the Debye–Waller (DW) factor (ratio of photons emitted in the zero phonon line (ZPL) to the overall spectrum, exchanging energy with phonons). The single photon lifetime (typically in the nanosecond scale and dependent on the transition dipole moment of the excited state) determines the rate at which the emitter is able to generate photons or process entanglement protocols (a photonic cavity could accelerate these time scales via the Purcell effect).

Aiming toward cryogenic-free applications, in this work, we study the single photon emission from defects in hBN nanocrystals at room temperature, extracting their two main dephasing mechanisms: the total spontaneous decay rate (γ/2π = 1/*T*_1_) and the pure dephasing rate (γ*/2π = 1/T*_2_, obtained in this work via Michelson interferometry). These two mechanisms contribute to the total dephasing rate and spectral line width of the emitter Γ = γ + 2γ*, where Γ/2π = 2/*T*_2_ is the full width at half-maximum (FWHM) of the ZPL.^18^

The value of Γ in certain hBN defect species has been studied via resonant spectroscopy of the ZPL as a function of temperature, showing a phonon broadening that scales as Γ ∼ *T* ^3^.^19–21^ Other defect species in hBN nanocrystals, similar to those studied in this work, display Γ ∼ *T* ^5^.^22^ Fourier transform limited line widths (Γ ∼ γ up to the 10 ms time scale) of certain hBN defects have been reported at room temperature.^23,24^ Later theory work on Density Functional Theory simulations for *C*_2_ *C*_N_ and *V*_N_ *N*_B_ defects confirm no decoupling effects from the phonon bath.^25^

At cryogenic temperatures, Fourier transform-limited emission is achievable for resonant ZPL scans within <10 μs, following on recent experiments with blue emitters (B-centers at 436 nm).^26,27^ For longer time scales, the temporal coherence is limited by inhomogeneous broadening arising from spectral diffusion. This effect is caused by fluctuations of the charge distribution around the environment of the emitter, and it is typically observed in solid-state color centers.^20^ Spectral diffusion in hBN at cryogenic temperature has been studied via photon-correlation Fourier spectroscopy,^28^ revealing the appearance of inhomogeneous broadening at different time scales (∼1 μs in ref 19. and ∼50 ns as well as longer time scales in ref 29.). Other techniques, such as femtosecond pump–probe spectroscopy have revealed that inhomogeneous broadening due to spectral fluctuations arises at scales as fast as ∼19 ps at cryogenic temperatures.^30^ The distinction between slow jitter components, such as the inhomogeneous broadening time and slow spectral jumps could be achieved via nonlinear spectroscopy techniques, such as four-wave mixing.^31^ Although some factors such as the sample preparation or the used substrate have proven to play an important role on inhomogeneous broadening, so far, no significant temperature dependence has been observed on this effect.^32^ Two-photon coalescence via Hong-Ou-Mandel interference, and off-resonant driving, has been reported for B-centers,^33^ determining a (temporally filtered) pure dephasing rate of γ*∼ 0.8γ for consecutively emitted photons with a delay of 12.5 ns. Under resonant driving, recient studies on B-centers have measured this two-photon interference, reporting in this case an indistinguishability value of 0.92 for the same delay between consecutive single photons.^34^ Temporal coherence of the single photon emission, under nonresonant excitation, is also characterized via Michelson interferometry. Following this method, and at cryogenic temperatures, the ZPL of hBN defects reveals a γ*∼ 60γ.^19^

Following this trend of results, our experiments, all implemented at room temperature, investigate the temporal coherence of hBN emitters in nanocrystals. In the first part of the work, we describe the fundamental emission properties of an hBN defect (spectrum, decay dynamics, and degree of photon antibunching). In the final part of the work, our experiments determine the pure dephasing time scale of this emitter and determine their dependence as a function of the wavelength of the excitation laser. We observe a phonon-induced pure dephasing time several orders of magnitude faster than the spontaneous emission lifetime and not a significant dependence on the laser energy.

## Methods

To prepare the sample, a commercial solution of hBN nanocrystals (Graphene Supermarket, with H_2_O as solvent) is drop-casted on a commercial distributed Bragg reflector (DBR). This is done without prior ultrasonic bath or postannealing process. The DBR mirror consists of 10 pairs of SiO_2_/Ti O_2_ layers with its stopband centered at 650 nm (1.907 eV). This substrate is used to enhance the collection efficiency for the studied spectral window. In future experiments, the DBR would be part of a Fabry-Pérot cavity for the study of cavity effects in the coherence properties of the emitters.^35,36^ The hBN nanometric crystals are randomly scattered over the sample, with ZPLs emitting in a wide energy range, between 560 and 750 nm (1.653–2.214 eV). We reconstruct the sample topography with scanning microscopic images to nanometrically locate defects at specific positions on the sample. The sample is navigated with XYZ closed-loop piezo-motors suitable for working at room temperature.

We implement microphotoluminescence (PL) experiments under nonresonant laser excitation (a 450 nm Q-switch laser operated in continuous wave (CW) or pulsed regime) in a home-built confocal microscope (see setup details in the Supporting Information). In the last part of our results, we also show experiments on several defects driven with not only 450 nm, but also 532 and 640 nm lasers. The single photon emission is collected using a 0.55 numerical aperture objective. In the collection path, the excitation laser is removed by a set of (tunable short- and long-pass) spectral filters. Then, the emission is sent to a spectrometer, or coupled into a single-mode fiber to perform time-resolved photoluminescence, Michelson interferometry (*g* ^(1)^(τ)) or Hanbury-Brown & Twiss correlation experiments (*g* ^(2)^(τ)), with different sets of fiber-coupled avalanche photodetectors (∼200/40 ps jitter time and high/low detection efficiencies, respectively).

In the Michelson interferometer, the mirror in the delay arm is attached to a piezoelectric actuator with a range of motion of 20 μm (corresponding to a fine-tunable delay of ∼133 fs). In addition, this assembly is mounted on a motorized translation stage with micrometric precision, which allows us to reach longer delays in the order of tens of picoseconds (maximum spatial displacement of 5 mm, ranging from −20 to 14 ps around zero delay, according to the relative positioning of the fixed and movable mirrors). The intensity resulting from the single-photon interference in the Michelson output is recoupled to a single-mode fiber and its count-rate is measured in a single photon detector versus temporal delay. A more thorough description of the Michelson set up is provided in the Supporting Information. The single-photon detection events (lifetime, and correlation measurements) are processed with the Extensible Time-tag Analyzer software tool.^37^

## Results

First, we study the PL spectrum of a single hBN emitter under nonresonant CW excitation in Figure 1(a). The ZPL is identified at 1.746 eV, presenting a FWHM of 5 meV. Two asymmetric shoulders surround the ZPL peak, these low-energy (LE) absorption and emission phonon modes correspond to longitudinal acoustic phonons and a localized vibrational mode.^38–40^ Although the exact structure of this defect is not conclusively determined, the ZPL emission could originate from different types of defects such as B-antisite, B-interstitial, or carbon substitutions in B/N vacancies.^22,41,42^ To distinguish the different spectral contributions present in the phonon sideband (PSB), we fit the experimental data to the sum of several Lorentzian functions (see Figure 1(a)), accounting for the emission coming from the ZPL, the longitudinal optical (LO) modes and the LE phonon modes. We obtain a DW factor of 0.77 ± 0.02 from the spectral fitting, and a LO contribution (to total emission) of 3%. These values agree with previous works providing an exhaustive analysis of the spectral properties of hBN emitters at room temperature.^41,42^ We evaluate an average DW factor of 0.43 ± 0.18 by studying 12 different emitters in this sample (see Supporting information). We note that the DW factor of the emitter shown in Figure 1 may be artificially enhanced by the fact that part of the PSB contribution lies in the limit of the DBR stopband, reducing its collection efficiency versus the ZPL (see Supporting information). In the second part of the work, we will study the temporal coherence of the ZPL and full spectrum regions. The low energy filtering region of the full spectrum used for this study is marked with a turquoise vertical dashed line, and the vertical dashed gray lines are used to indicate the filtered ZPL region.

**Figure 1:**
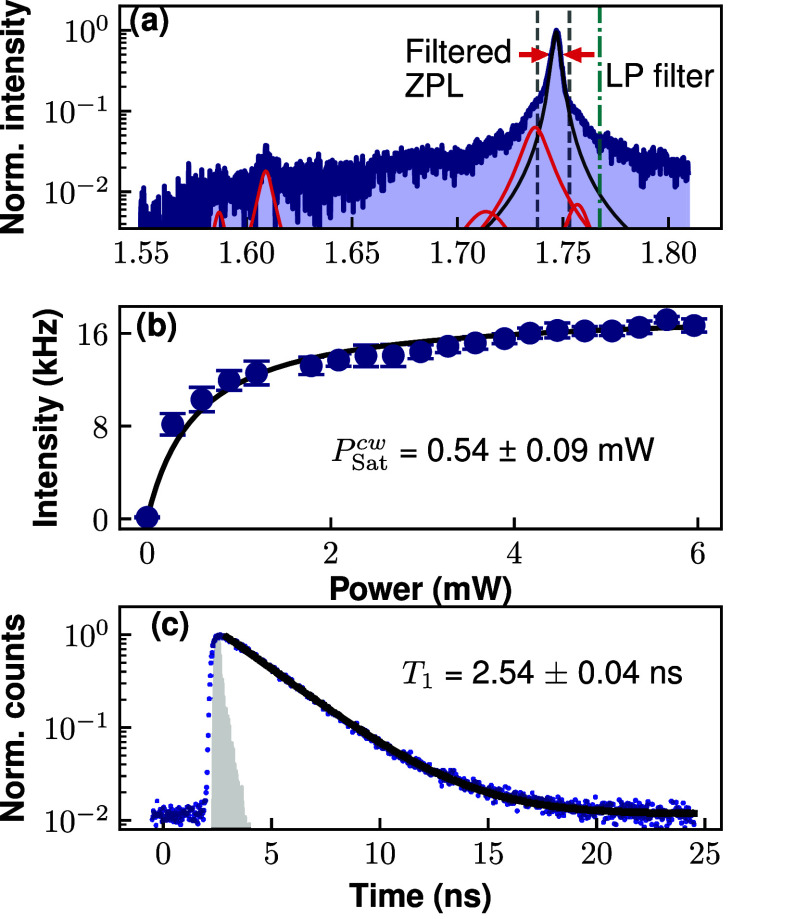
Spectral and temporal characterization of the hBN emitter at room temperature. (a) PL spectrum in log-scale under CW, nonresonant (450 nm) laser excitation, and 1.85 *P*_Sat_^CW^ excitation power. The ZPL (black Lorentian fit) is located at 1.747 eV, while the rest of the emission (red Lorentzian peaks) comes from the PSB. The red arrows indicate the hBN defect spectrum FWHM. The vertical dashed lines indicate the filtered spectrum of the ZPL (gray) and the full spectrum (turquoise low-energy band-pass) subsequently analyzed in the Michelson interferometer. (b) ZPL pump power dependence, recording the intensity with a single-photon detector, *P*_Sat_^CW^= 0.54 mW. (c) Spontaneous decay of the emitter showing a monoexponential decay *T*_1_ = 2.54 ± 0.04 ns, measured under a pump power of 1.2 *P*_Sat_^p^. The instrument response function of the detector is included in a gray-shaded area.

We continue studying the pump power dependency of the ZPL emission under CW excitation. In Figure 1 (b), the excitation power dependent intensity of the filtered ZPL is fitted with the function *I*(*P*) = *I*_∞_^cw^/(1 + *P*_Sat_^cw^/*P*), which models the saturation dependence of a two-level system under incoherent excitation.^43^ The saturation power is *P*_Sat_^cw^ = 0.54 ± 0.09 mW. The value of *I*_∞_^cw^ = 18.0 ± 0.4 kHz indicates that the source-to-detector efficiency (*B*_d_, which includes the setup and detection inefficiency), in units of the emitter lifetime (*T*_1_ see below in Figure 1(c)) is *B*_d_ ∼ *I*_∞_^cw^ *T*_1_ = 0.005%. The same saturation curve (see Supporting Information) is measured under pulsed excitation, obtaining *I*_∞_^*p*^ = 3.2 ± 0.1 kHz and *P*_Sat_^p^ = 42 ± 3 μW; in this case, *B*_d_ ∼ 0.008%. The small difference between these CW and pulsed source-to-detector efficiency values may arise from a different setup performance (fiber-coupling) for these two experiments and different emitter blinking behavior in each driving regime.

The ZPL defect lifetime is *T*_1_ = 2.54 ± 0.04 ns, as obtained from the monoexponential fit shown in Figure 1(c); the instrument response function of the fast photon detector is indicated in a gray filled area. From the *T*_1_ value, we derive a Fourier-limited line width of Γ_FL_/2π = 62.7 MHz, several orders of magnitude narrower than the ZPL line width: the ZPL is strongly broadened due to electron–phonon coupling processes.^22^ The ZPL lifetime of other defects (not shown here) display similar values in the order of a few nanoseconds. The power-dependence and lifetime measurements are recorded with a low-jitter single photon detector, with an efficiency of <30% and ∼40 ps jitter time.

To confirm the single photon character of the defect emission, we measure the second order correlation function *g* ^(2)^(τ) via a Hanbury-Brown and Twiss setup, under both pulsed and CW excitation. In pulsed regime, we obtain a value of *g* ^(2)^(0) = 0.11 ± 0.01 under 1.2 *P*_Sat_^*p*^, see Figure 2(a). Due to the emitter blinking, we note that the peaks near zero delay (not used for the *g* ^(2)^(0) normalization) present a bunching four times more intense than the uncorrelated peaks at long delays (the gray horizontal line in this panel shows the average height of the peaks for |τ|∼ 1 μs). Although we do not discuss it here, we observe a worsening of the pulsed *g* ^(2)^(0) value as the pulsed pump power increases, which arises from re-excitation processes during the laser pulse.

**Figure 2:**
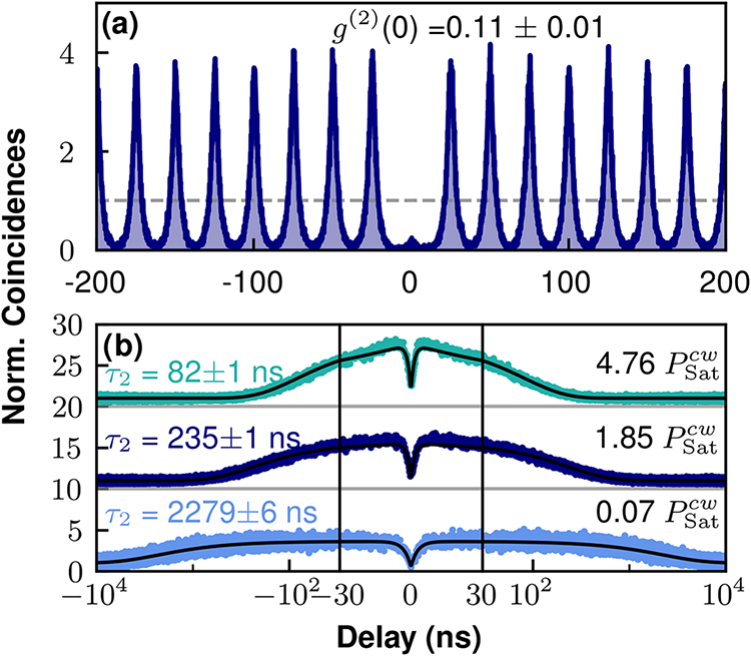
Single photon character and blinking of the hBN emission. (a) Pulsed second-order correlation function under low pump power excitation, 1.2 *P*_Sat_^p^ laser power and 40 MHz repetition rate. The measured antibunching is *g* ^(2)^(0) = 0.11 ± 0.01 (this result does not account for the two-detector jitter). The horizontal, dashed line marks the average height of uncorrelated peaks at long delays. (b) CW second-order correlation for different pumping powers. Similarly to panel (a), the histogram normalization is done with the uncorrelated coincidence peaks at long-delays. The correlation curves are vertically displaced for clarity (the horizontal black lines at 10 and 20 normalized coincidence levels mark the correlation baseline for the medium and high driving powers). The bunching times τ_2_ are specified in the left side of the panel.

Under CW excitation and weak (0.07 *P*_Sat_^cw^) pump power, we measure *g* ^(2)^(0) = 0.46 ± 0.13; similarly, this value is normalized with the uncorrelated peaks at long delay and without accounting for the two-detector jitter time (∼ 200 ps per detector). We note that this value is significantly larger than the *g* ^(2)^(0) measured under pulsed excitation with higher pump power (in relation to their corresponding *P*_Sat_). We attribute this difference in antibunching to the slow temporal resolution of the detectors, compared to the antibunching time scale τ_1_ around zero delay. This CW *g* ^(2)^(τ) has also been studied for 1.85 *P*_Sat_^cw^ and 4.76 *P*_Sat_^cw^ to observe the power-dependent blinking dynamics (see Figure 2(b)). For low excitation power, there is a weak bunching effect at microsecond time scales. When pump power is increased, this time scale is reduced from 2.28 μs (0.07*P*_Sat_^cw^) down to 0.08 μs (4.76*P*_Sat_^cw^) and the bunching amplitude is increased. Such a blinking behavior is a typical signature of the presence of a dark state in a three-level ladder, affecting the emitter brightness.^44^

Due to re-excitation processes under CW driving, the antibunching time scale, τ_1_, decreases for higher pump powers. While for the lowest excitation power (0.07 *P*_Sat_^cw^) this value is similar to *T*_1_ (τ_1_^low^ = 2.78 ± 0.10 ns), it is notably shorter for medium (τ_1_^med.^ = 1.49 ± 0.04 ns) and high (τ_1_^high^ = 1.31 ± 0.03 ns) drivings (see antibunching dips in Figure 2(b)). For the correlation histograms with 1.85 *P*_Sat_^cw^ and 4.76 *P*_Sat_^cw^, τ_1_ is close to the detectors jitter time and the blinking bunching is very prominent. In these conditions, our slow detectors can not resolve the antibunching dip and therefore the *g* ^(2)^(0) appears overestimated.

The mean-wavepacket overlap, i.e., indistinguishability, of the emitted single photon is determined by the ratio γ/Γ (or equivalently *T*_2_/(2*T*_1_)). Apart from the ZPL resonant PL scan (which does not account for dephasing mechanisms occurring under nonresonant excitation of the defect), a precise measurement of Γ can be obtained via Michelson interferometry.^19,45,46^ This measurement provides the total dephasing time of the emitted single photon along its lifetime time scale, as compared to a two-photon coalescence experiment, which captures dephasing in the time scale of the delay between two successively emitted single photons.^33^ eq 1 shows the expected single-photon intensity (*N*_out_) measured in the output interferometer arm, assuming that the emitter spectrum is Lorentzian and the two interfering modes perfectly overlap in the central beam splitter.

1 $$N_{\mathrm{out}}=\frac{1}{2}\left(1+e^{-\Gamma /2\tau }\cos\left(\omega_{o}\tau \right)\right)$$

In this expression, Γ determines the exponential decay of the fringe amplitude as a function of the delay between optical paths τ (ω_0_ is the frequency of the Lorentzian peak). As shown in the following, our room temperature experiments set the phonon bath as the main source of decoherence, in a regime where γ* ≫ γ.

Figure 3 compiles our experiments on the temporal coherence of the single photons emitted from the defect under study. We analyze the pure dephasing rate of the filtered ZPL spectrum (dark blue symbols) and the full spectrum filtered with just a long-pass filter (turquoise data points, see filtered spectrum from Figure 1(a)). In the case of the filtered ZPL, the Lorentzian fits in Figure 1(a) show that ∼92% of the intensity comes from the ZPL and ∼ 8% from LE phonon modes, whereas contributions from other phonon modes represent less than 1%. The pump power used for these experiments is 1.85 *P*_Sat_^cw^. For the sake of clarity, Figures 3(b–d) show the interference fringes for different time delays (see time scale in the horizontal axis for each panel). Every point in panel (a) corresponds to the amplitude visibility calculated for a single oscillation period of *N*_out_.

**Figure 3:**
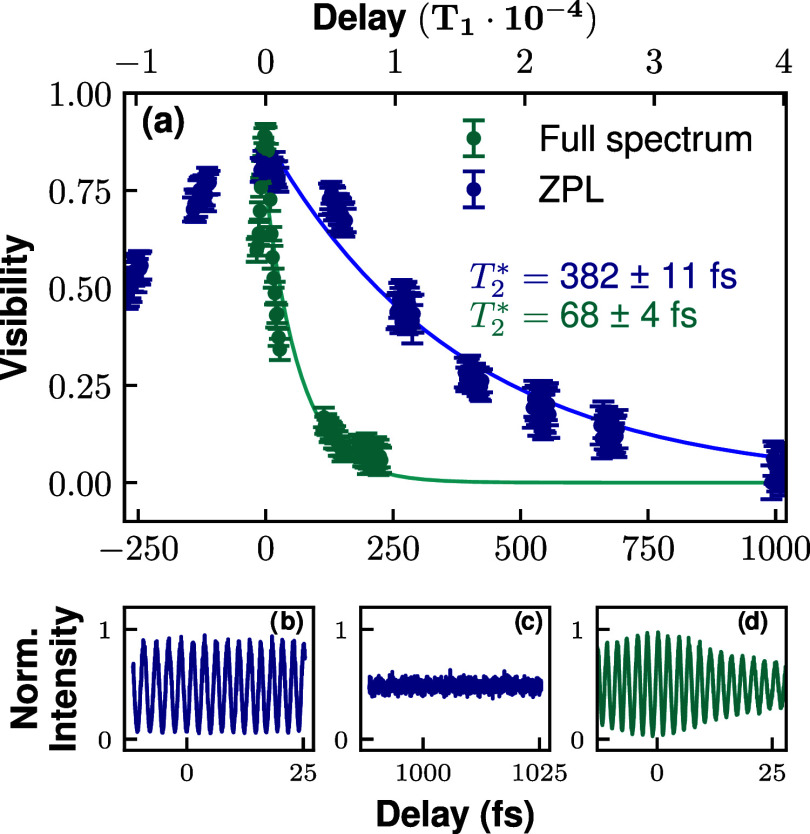
Temporal coherence via Michelson interference. (a) Fringe visibility in the output mode of the Michelson interferometer as a function of the temporal delay between the two arms for the filtered ZPL (dark blue trace) and full spectrum (turquoise). The portion of the spectrum used for each data set is indicated in Figure 1(a). Panels (b–d) show the normalized intensity oscillations as a function of the piezo-tuned fine delay.

Both sets of data (ZPL and full spectrum) are fitted with the exponential decay given in eq 1; the corresponding pure dephasing times resulting from the fits are *T*_2_^*^ = 382 ± 11 fs for ZPL (dark blue trace), and 68 ± 4 fs (turquoise trace) for the whole spectrum. In the next section we discuss the dependence of the coherence time on the filter width. We note that, at zero delay, the maximum visibility of the ZPL is ∼80%, indicating that the spatial mode overlap of the beams interfering in the beam splitter is not perfect.

Next, we extend our measurements to study the effect of the driving energy on the coherence time of hBN single photon emission. We observe that most of the defects found with the 450 nm (blue) laser do not emit when excited with the 532 nm (green) or the 640 nm (red) laser, since there are fewer available phonon-assisted processes to drive the defect at smaller detunings. Figure 4(a,c,e) show the PL spectra of three emitters (dubbed I, II and III, respectively, as labeled in the figure) that are successfully excited using more than one driving energy. Overall, only small differences appear when we compare the spectra, apart from a notable change in the emission intensity. Under the given experimental conditions, we observed different excitation energy dependency in the DW factor. While emitter I increases its DW factor with lower detuning excitation, emitters II and III display the opposite behavior. In the case of emitter I, the prominent LE phonon mode, present for the blue driving, disappears with green excitation. Regarding the spectral properties of emitter III, it presents a broader ZPL (FWHM ∼ 8 meV) but very similar energy to the emitter characterized in Figure 1. It also displays a very weak LO phonon band, coherent with the description of ref 41. However, LE phonon modes are more important for emitter III, which makes its DW factor considerably lower, especially for the red laser excitation.

**Figure 4:**
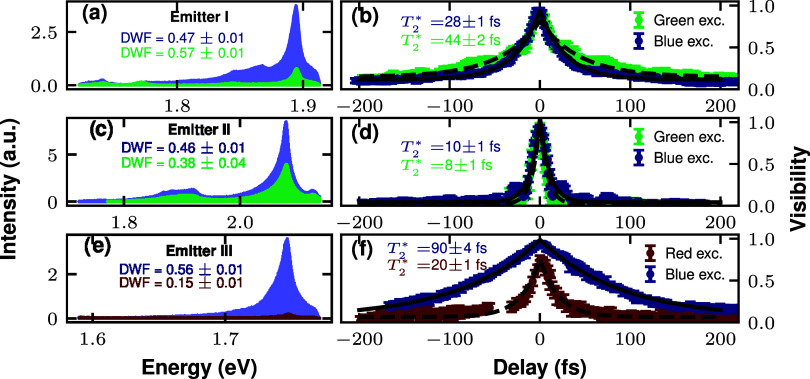
Temporal coherence under different excitation energies. Left panels (a, c, e) show the PL spectra of three different emitters (that we call emitter I, II and III respectively) driven with two excitation energies for each case. Right panels (b, d, f) display the corresponding visibility decay for the Michelson interference performed for the defects and excitation lasers on the left. Blue, green and red colors on the figure correspond with excitation energies of 2.755, 2.330, and 1.907 eV respectively. All the *T*_2_^*^ have been calculated from the exponential fits shown in the figures. Spectra and visibility curves have been extracted with CW excitation and power of 1 mW.

To check whether the driving energy makes a difference in the coherence properties of the emission, we perform Michelson interferometry for these defects. Figures 4 (b,d,f) show the corresponding visibility decay of emitters I, II and III (see driving conditions in the figure), respectively. Interestingly, we observe that longer *T*_2_^*^ correlates with higher DW factor. For instance, emitter I exhibits a longer *T*_2_^*^ of 44 ± 2 fs (green curve in Figure 4(b)) as the green laser excitation improves the DW factor by 0.1 compared to the blue excitation in Figure 4(a). The change in coherence time is especially relevant for the case of emitter III. In this case, *T*_2_^*^ is 90 ± 4 fs for blue excitation (see exponential fit in Figure 4(f)), and it reduces to 20 ± 1 fs for red laser driving. We note that in this case, the detuning of the red laser (∼159 meV) lies slightly below the 1 LO phonon excitation window, which is around 165 meV.^41^

## Discussion

There are several processes that govern the decoherence of solid-state single-photon emitters (such as electron, phonon and spin nuclei dephasing mechanisms). At low temperature, the spectral diffusion from charge fluctuations around the defect environment is the main factor for dephasing in hBN defects, as confirmed by the resonant ZPL excitation experiments of refs 20,21,26,27 This inhomogeneous broadening typically increases the spectral line width from ∼60 MHz up to ∼1 GHz. Decoherence processes in hBN defects at cryogenic temperatures (8–80 K) have also been studied via femtosecond pump–probe spectroscopy.^30^ Spectral jitter from homogeneous and inhomogeneous sources is identified producing an exponential and Gaussian dephasing broadening, respectively. The homogeneous dephasing time (from phonons) decreases from 55 ps at 8 K down to 3.5 ps at 80 K, with a combined emitter coherence time of 21 ps at 8 K. In this case, the inhomogeneous dephasing arises from two-mode spectral random jumps (picosecond scale periodicity), and slower (nanosecond scale and beyond) spectral shifts. In our experiments, we probe the ultrafast time scales (below 1 ps delays, see Figure 3), where phonon-induced homogeneous dephasing dominates over any other slower (1 ns and beyond) inhomogeneous source of decoherence, as the spectral broadening increases rapidly with *T* ^5^.^22^

Previous works at room temperature, based on a statistical description of the spectral properties of hBN defects, provide estimations for the ZPL coherence time of ∼100 fs for various families of emitters.^41^ We include two tables in the Supporting Information where coherence time values reported in refs 41,42 are discussed. The measurement of the dephasing time via Michelson interference provides a complete picture of the emitter dephasing mechanisms (also allowing for a spectrally selective analysis of the decoherence time); this is a particular advantage when the emitter spectrum presents a structure composed by ZPL and several phonon modes, as it is the case shown in Figure 1(a) (see LO and LE modes), also reported in other works.^36,47,48^

Coherence properties of emitters in different materials have been characterized in other works, typically at cryogenic temperatures. NV centers in diamond reveal a value of *T*_2_ ∼ 4.9 ps for the filtered ZPL (13 fs for the full spectrum), 4 orders of magnitude faster than its spontaneous decay time.^46^ Similarly, chromium centers in diamond present coherence times of ∼62 ps at 1.6K, which is 3% of the corresponding lifetime.^49^ In self-assembled quantum dots (QDs), coherence measurements (at cryogenic temperatures) via Michelson and photon-correlation Fourier spectroscopy show values for *T*_2_ ranging from few picoseconds (∼10 ps for InP QDs^50^) up to 770 ps for an InGaAs QD.^51^ At room temperature, nickel-based color centers in diamond show a *T*_2_ ∼ 210 fs.^47^ This result is close to our reported coherent times for hBN defects, operated under similar conditions. We include a table in the Supporting Information where these coherence times for different platforms are discussed.

As observed in Figure 3(a), the restrictive spectral filtering of the ZPL (as marked in Figure 1(a)) artificially increases the coherence time and makes the temporal shape of the visibility decay to be Gaussian like, as a direct consequence of the Wiener–Khintchine theorem. We corroborate this spectrum filtering effect by simply performing the Fourier transform of the measured filtered ZPL spectrum, retrieving a very similar dephasing time as that recorded via Michelson interferometry (see Supporting Information, Figure S6.)

It is important to note that the inhomogeneous dephasing mechanisms are too slow to take part in the Gaussian-shape visibility decay observed in the filtered spectrum of Figure 3(a). The temporal coherence is lost beyond 1 ps delay due to phonon-coupling at room temperature. The inhomogeneous broadening arises in the ∼10 ps time scale,^19,30^ and even longer (∼10 ns and ∼1 μs scales).^29^

From the values of the ZPL pure dephasing time, the probability of emitting two consecutive, coherent single photons under saturation conditions is ∼0.015%. Similarly, we can expect an upper bound (i.e., assuming 100% brightness) for the probability of two-photon interference in a path-delayed Mach–Zehnder interferometer. With a delay of consecutive single photons of 25 ns, this value is ∼0.0015%. This result indicates that the strong electron–phonon coupling requires working at cryogenic temperatures. We also note that the defect lifespan is rather short in our samples (ranging between days and a few weeks); in our case, such short lifespans may be attributed to the high energy detuning between the excitation laser and the red-detuned hBN emitter spectra under study especially under blue laser excitation (between 1.7 and 2 eV). Previous studies in similar defects have reported similar a behavior and have argued that power-dependent optically induced local charge fluctuations^52^ or energy-dependent photochemical reactions^53^ (particularly when these defects are close to the hBN surface) might be involved. We have also studied the ZPL Michelson visibility under different pump powers, observing almost identical dephasing times (not shown here), which allows us to discard pump power induced dephasing mechanisms versus the action of phonons under our experimental conditions.

Regarding the results for different driving energies shown in Figure 4, we can conclude that the use of very high detunings does not generally worsen the coherence time at room temperature. In this condition, the energy of excitation can be optimized considering the brightness and purity of single photon emission as the main criteria. Emitter III shows that the blue non resonant driving results in a larger coherence time that the red color laser. This wavelength-dependent behavior may involve distinct phonon-assisted excitation pathways or local photocharge effects. Further systematic experiments versus the excitation laser wavelength will be required to clarify its origin.

## Conclusion

We have characterized the pure dephasing rate of the single photon emission from hBN defects at room temperature via Michelson interferometry. This dephasing time (*T*_2_^*^) is of the order of a few hundred femtoseconds, 4 orders of magnitude faster than the spontaneous decay time (*T*_1_), due to the strong electron–phonon coupling mechanisms. Consequently, such room temperature single photon emission constrains the quantum photonic application landscape to protocols where photon interference is not required, such as BB84 ^54,55^ and B92 ^56,57^ or random number generation protocols.^58,59^

In following experiments, the single photon emission from these defects will be studied at lower temperatures to attenuate the phonon dephasing rate on the temporal coherence of the emission. Coupling the defect to a photonic cavity at cryogenic temperatures will further reduce the spontaneous decay lifetime via the Purcell effect in comparison to the environmental charge fluctuations dynamics, still present at these temperatures.^20–22,26,27^ Provided the large range of energies where hBN defects are present,^60^ we believe that a reconfigurable, open Fabry-Pérot cavity may be a suitable architecture to expand the potential applications across the visible and near-infrared bands, producing efficient and coherent single photons.^61^

## Supplementary Material



## Data Availability

The data underlying this studies are openly available in the repository e-cienciaDatos at 10.21950/OSZVZO.

## Notes

Experimental setup, sample substrate: DBR mirror transmitance, saturation curve under pulsed driving, spectral decomposition of some emitters, Fourier Transform of the filtered ZPL spectrum, comparison of the coherence time of other single photon emission platforms (PDF)
