# Supplementary material for: Temporal Coherence of Single Photons Emitted by Hexagonal Boron Nitride Defects at Room Temperature
Source: ACS Photonics. 2025 Dec 27;13(1):282–9. doi: 10.1021/acsphotonics.5c02227 (PMC12784394; doi:10.1021/acsphotonics.5c02227)
Supplement: Supplementary file 1 [file ph5c02227_si_001.pdf]

Supporting information: Temporal coherence of single photons emitted by hexagonal Boron Nitride defects at room temperature.

J.-V. Vidal Martínez-Pons,[1, 2, *] S.-K. Kim,[3] M. Behrens,[1] A. Izquierdo-Molina,[1] A. Menendez Rua,[1] S. Paçal,[4] S. Ateş,[5] L. Viña,[1, 2, 6] and C. Antón-Solanas[1, 2, 6, †]

[1] *Depto. de Física de Materiales, Universidad Autónoma de Madrid, 28049 Madrid, Spain.*
[2] *Instituto Nicolás Cabrera, Universidad Autónoma de Madrid, 28049 Madrid, Spain.*
[3] *Walter Schottky Institut, Institute for Advanced Study, TUM School of Computation, Information and Technology, and MCQST, Technische Universität München, 85748 Garching, Germany.*
[4] *Department of Physics, Izmir Institute of Technology, Izmir 35430, Turkey.*
[5] *Faculty of Engineering and Natural Sciences, Sabanci University, 34956, Tuzla, Istanbul, Turkey.*
[6] *Centro de Física de la Materia Condensada (IFIMAC), Universidad Autónoma de Madrid, 28049 Madrid, Spain.*

CONTENTS

I. Experimental setup S2

II. Sample substrate: DBR mirror transmitance S3

III. Saturation curve under pulsed driving S3

IV. Spectral decomposition of some emitters S3

V. Fourier transform of the filtered ZPL spectrum S4

VI. Comparison of the coherence time of other single photon emission platforms S5

References S6

* juan.vidal@uam.es
† carlos.anton@uam.es

I. EXPERIMENTAL SETUP

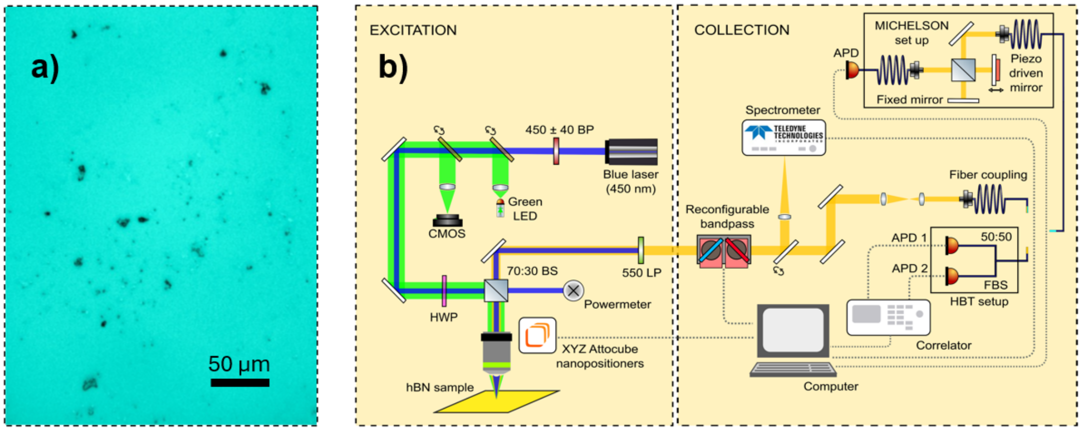

FIG. S1. (a) Microscope image of the hBN drop-casted on the DBR substrate. (b) Experimental setup for the study and characterization of emission from hBN color centers. The excitation line includes a 450 nm laser and a LED+CMOS camera to visualize the sample. The objective and sample are mounted on Attocube closed-loop nanopositioners that allow total control in the XYZ directions. In the collection line, the excitation laser is filtered out with a 550 nm LP and an additional filtering level can be added with a reconfigurable bandpass, allowing a band selection between 615-710 nm with minimum FWHM of 2 nm. The filtered emission can be studied in a spectrometer or be fiber coupled. The fiber-coupled light can be sent to study the purity of individual photons with a Hanbury-Brown & Twiss setup or the temporal coherence with a Michelson interferometer.

A confocal microscopy setup is used at room temperature for non-resonant excitation and collection of the photoluminescence from hBN defects (see Fig. S1). A CMOS camera and a 530 nm green LED are used to illuminate and image the sample surface in order to locate potential defect sites. The sample is mounted on a set of XYZ closed-loop Attocube nanopositioners, which allow precise positioning in three dimensions.

Excitation is performed using a 450 nm PicoQuant Q-switch laser which can be operated in continuous wave or pulsed regime (with pulse length of ∼70 ps). A half-wave plate is inserted into the excitation path to control the polarization of the laser beam, enabling optimization of the detected photon counts. A 70:30 (transmission:reflection) beam splitter is used to direct the excitation beam towards the sample while simultaneously allowing the emitted photons to propagate into the collection path. The laser is focused on the sample with a Mitutoyo M Plan APO 50x objective (N.A. = 0.55), whose Z-axis can be controlled by a New Focus micro-positioner.

The emitted light is filtered using a set of short- and long-pass tunable 704 nm Semrock filters mounted on independent motorized rotation stages. By removing a flip mirror, the collected emission can be directed either to a spectrometer or coupled into an optical fiber. The fiber-coupled light can then be sent to either a free-space Michelson interferometer for coherence measurements or a fiber-based Hanbury Brown and Twiss (HBT) setup for photon correlation analysis.

The Michelson interferometer consists of a 50:50 beam splitter, with one output directed to a stationary mirror and the other to a mirror mounted on a piezoelectric actuator. The piezo actuator is attached to a motorized translation stage, enabling coarse movement over millimeter-scale distances, while the piezo actuator provides fine nanometer-level control. One of the two outputs is coupled again in a single-mode fiber in order to measure the intensity in an APD.

To measure the single-photon self-interference with this setup, we proceed as follows. The piezo (range 20 μm, maximum voltage 75 V) is driven with a periodic triangular electric signal between 0.5 and 9.5 V, which is amplified by a 7.5 factor. The repetition rate of this signal is 10 Hz. The same signal generator provides a CMOS-shaped signal with the same repetition rate which is used to trigger the correlator. Then, the number of detection events by the APD is represented as a function of delay with the trigger signal. The relation between the time delay of the electric signals (APD and trigger) and the path difference (or equivalently the delay τ) is calculated by using the periodic oscillations as a reference. The closed loop motorized stage (5 mm range) can be moved to reach longer delays. We integrated over ∼5 million events for each induvidual piezo scan.

II. SAMPLE SUBSTRATE: DBR MIRROR TRANSMITANCE

The hBN crystals are deposited on a DBR mirror with 10 pairs of SiO_2/TiO_2 layers, purchased in Laseroptik. The transmission curve for this substrate is shown in Fig. S2. As mentioned in the main text, the ZPL lies inside the stopband, but part of the PSB is out of it.

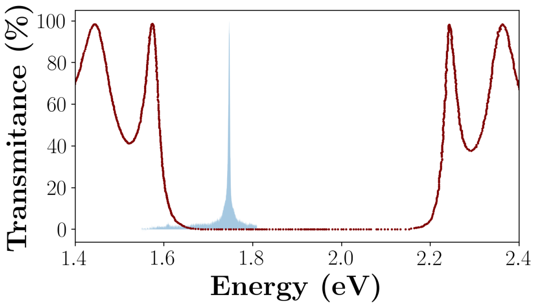

FIG. S2. Transmission spectrum of the DBR substrate used to dropcast the hBN solution. The spectrum of the defect studied in the main text is also shown in blue.

III. SATURATION CURVE UNDER PULSED DRIVING

Fig. S3 shows the characteristic two-level system saturation curve under pulsed excitation with 40 Hz repetition rate. The emitter reaches half of its maximum intensity (I^p_∞=2.70 ± 0.03 kHz) at 0.042 ± 0.003 mW. The detected brightness, calculated as the ratio between detected events and laser pulses, is 0.007%.

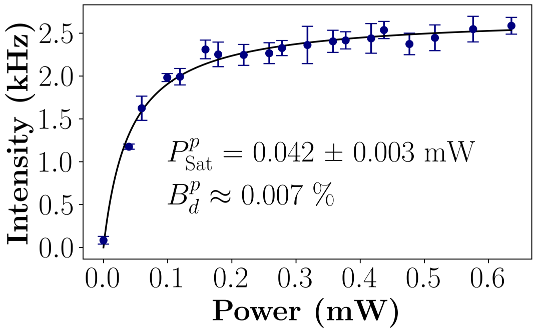

FIG. S3. Pump power saturation curve under pulsed excitation, the value of the saturated intensity is I^p_∞=2.70 ± 0.03 kHz and the value of the pulsed saturation power is $P^p_{\rm Sat} = 0.042 \pm 0.03$ mW.

IV. SPECTRAL DECOMPOSITION OF SOME EMITTERS

To extract the DW factor of the emitter shown in the main text, we have fitted the experimental spectrum with seven Lorentzian peaks: ZPL, two longitudinal optical phonon modes for detunings of 165 meV and 195 meV, and two acoustic modes at 14 meV and 30 meV (in absorption and emission). A similar analysis has been carried out for other defects within the sample (see Table S1 and S4).

Emitter	ZPL wavelength (nm)	Excitation wavelength (nm)	ZPL linewidth (meV)	A_{LO}/A_{ZPL}	A_{LE}/A_{ZPL}	$A_{LE(abs)}/A_{ZPL}$	Debye-Waller factor
(a)	571.9	450	8.4	0.14	0.71	0.04	0.53±0.02
(b)	577.7	450	6.7	0.45	1.93	0.85	0.24±0.01
(c)	581.9	532	16.8	1.01	0.65	0.29	0.34±0.01
(d)	596.3	450	15.0	0.46	0.65	0.09	0.46±0.01
(e)	619.8	450	8.3	1.11	1.95	0.55	0.22±0.01
(f)	627.7	450	7.9	1.84	2.03	1.42	0.16±0.02
(g)	629.5	450	6.3	0.67	0.75	<0.01	0.41±0.01
(h)	648.4	532	11.8	0.10	0.29	0.17	0.64±0.03
(i)	653.8	450	7.4	0.19	0.78	<0.01	0.51±0.02
(j)	660.7	532	8.4	0.55	1.33	0.32	0.31±0.01
(k)	709.1	450	7.9	0.11	0.55	0.12	0.56±0.01
(l)	709.7	450	4.1	0.04	0.24	0.02	0.77±0.02

TABLE S1. Spectral characteristics of the emitters presented in Fig. S4. All values have been extracted from the Lorentzian fits.

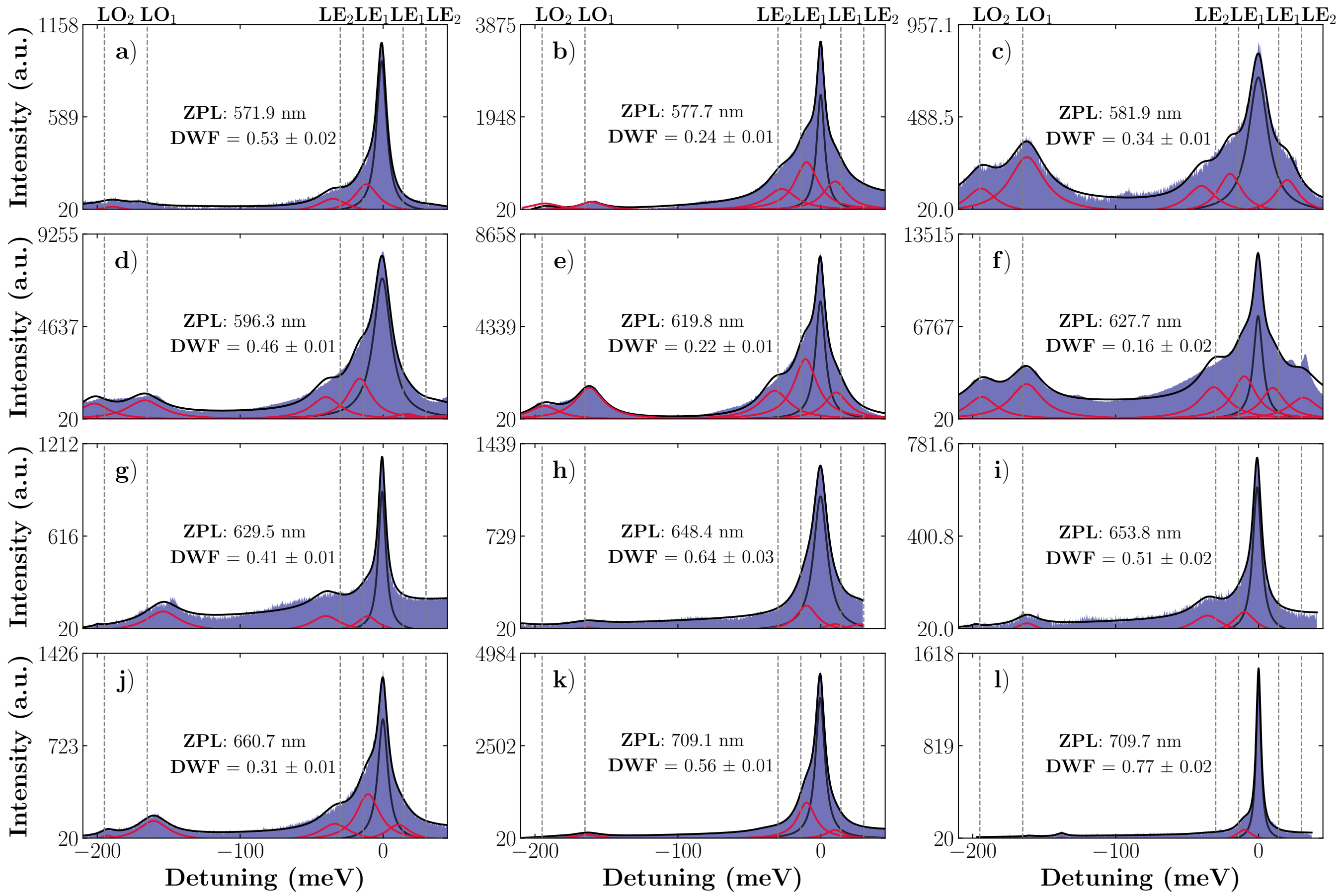

FIG. S4. Spectra of 12 different emitters. The red Lorentzian fits correspond to the contributions of the phonon bands, whereas the black line accounts for the sum of all contributions. The experimental data is represented by the blue shade. The vertical lines indicate the typical energies of each phonon mode.

V. FOURIER TRANSFORM OF THE FILTERED ZPL SPECTRUM

The spectral distribution of the emission and the $g^1(\tau)$ form a Fourier-transform pair. Hence, for a Lorentzian distribution we expect an exponentially decaying visibility of the Michelson interference, but we expect a different decay if the emission has other spectral shape. We have performed the Fourier transform of the spectral distribution

of the filtered ZPL to confirm that our experimental $g^1(\tau)$ matches the expected visibility profile. The result is shown in Fig S5. The Fourier transform has been normalized so that the maximum coincides with the maximum visibility measured in our experiment. Similarly, we have simulated the effect of spectral filtering on the visibility measurements (see Fig. S6), finding that the shape shown in Fig. S5 disappears when the filtering region becomes broader.

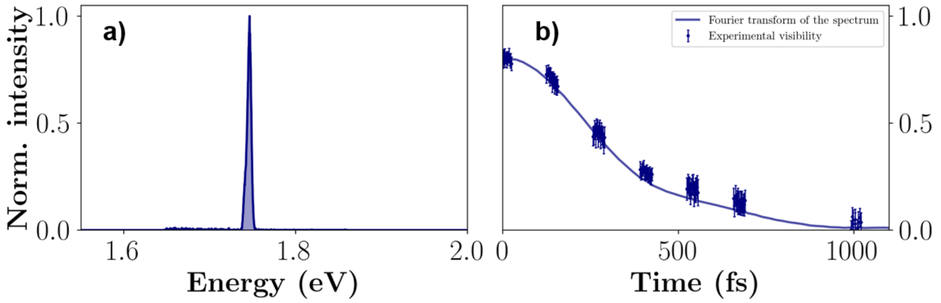

FIG. S5. (a) Normalized emission spectra filtered. (b) Fourier Transform of spectrum after normalization. Dots correspond to experimental visibility of the Michelson interference.

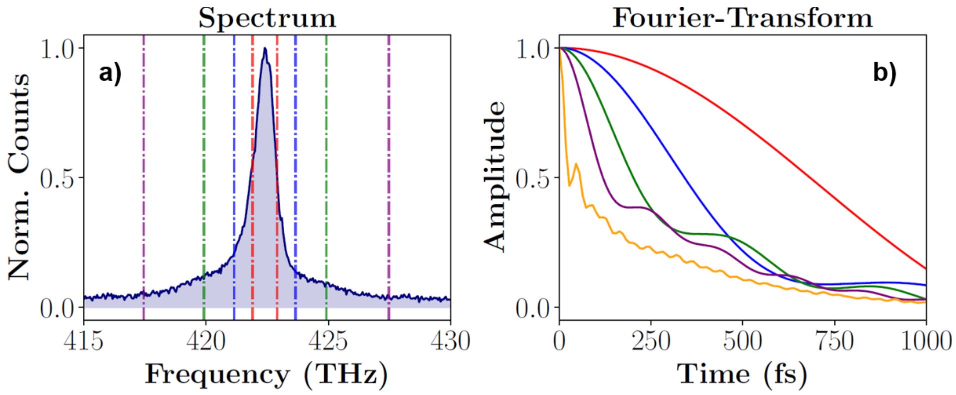

FIG. S6. Different spectral filtering windows (a) and their corresponding Fourier transform (b). The yellow curve on the right corresponds to a filter choice (400-450 THz, we keep theis scale in panel (a) for the sake of a direct comparison with the Fourier transformed panel (b)) wider than the spectral region shown in panel (a). The use of a narrow spectral window around the ZPL (red filtering) increases the decay time of the corresponding FT and makes it Gaussian-like rather than exponential (see red curve in panel (b)).

VI. COMPARISON OF THE COHERENCE TIME OF OTHER SINGLE PHOTON EMISSION PLATFORMS

In this section, we provide a compilation of previous measurements on coherence properties of various platforms for single-photon emission. Tables S2 and S3 correspond to statistical description of hBN emitters presented in Refs [1] and [2] respectively. All the estimations of coherence times are based just on spectral measurements. Table S4 show a summary of coherence measurements results of other platforms via Michelson interferometry or photon-correlation Fourier-spectroscopy.

Ref. [1]	(a)	(b)	(c)	(d)	(e)	(e)	(e)	(e)
Temp. (K)	300	300	7	7	300	150	50	20
E_{ZPL}(eV)	2.127	2.254	1.969	2.096	2.134	2.136	2.138	2.139
T_2^{ZPL}(ps)	0.08	0.12	0.8	0.8	0.17	0.33	0.4	0.5

TABLE S2. Compilation of ZPL energies and T_2^{ZPL} values from Ref. [1]. The cases measured at room temperature are highlighted in grey color. The labels (a-e) follow the same nomenclature as that used in Ref. [1].

Defect families in Ref. [2]	F_1	F_2	F_3	F_4	F_5	F_6	F_7	F_8	F_9	F_{10}	F_{11}
E_{ZPL}(eV)	2.01	1.96	1.91	1.86	1.85	1.83	1.79	1.77	1.74	1.69	1.63
HWHM$_{ZPL}$ (meV)	10.2	11.3	10.5	9.3	10.8	9.9	3.6	8.7	7.8	10.1	9.2
T_2^{ZPL} from spectrum (ps)	0.021	0.019	0.02	0.023	0.019	0.021	0.058	0.024	0.027	0.021	0.023

TABLE S3. Compilation of ZPL energies and T_2^{ZPL} values from Ref. [2], which have been derived from the FWHM of the spectra; we identify our defect under study as part of family F_9 (highlighted in grey color).

Platform	Ref.	E_{ZPL} (eV)	Temp. (K)	T_1 (ns)	T_2 (ps)	T_2^{ZPL} (ps)
NV center	[3]	1.945	1.6	10	0.013	4.9
Cr center	[4]	∼1.76	4	1.7	-	62[A], 57[B]
Nickel center	[5]	1.561	300	1.5	-	0.21
Perovskite QDs	[6]	∼2.37	3.6	∼270	-	∼50-80
InP QDs	[7]	1.81	7	1.2	-	∼7-17
InGaAs QDs	[8]	1.332 [QD1]	5	1100	770	-
GaAs QDs	[9]	1.589 [X]<br>1.585 [XX]	5	267<br>115	After 2 ns<br>167 [X]<br>105 [XX]<br>After 10 ms<br>22 [X]<br>40 [XX]	-
InGaAs QDs	[9]	1.390 [X]<br>1.388 [XX]	5	351<br>186	After 2 ns<br>165 [X]<br>103 [XX]<br>After 10 ms<br>87 [X]<br>78 [XX]	-
WSe_2 QDs	[10]	1.608	4	2.4	-	13.5

TABLE S4. Compilation of T_1 and T_2 values for several single photon emission platforms under different temperature conditions. Specific emitters in Refs. [14] and [18] are cited following the corresponding work nomenclature. The notation X and XX in Ref. [19] stands for neutral exciton and biexciton, respectively.

[1] D. Wigger, R. Schmidt, O. Del Pozo-Zamudio, J. A. Preuß, P. Tonndorf, R. Schneider, P. Steeger, J. Kern, Y. Khodaei, J. Sperling, et al., 2D Materials **6**, 035006 (2019).

[2] M. S. Islam, R. K. Chowdhury, M. Barthelemy, L. Moczko, P. Hebraud, S. Berciaud, A. Barsella, and F. Fras, ACS nano **18**, 20980 (2024).

[3] F. Jelezko, A. Volkmer, I. Popa, K. Rebane, and J. Wrachtrup, Physical Review A **67**, 041802 (2003).

[4] T. Mueller, I. Aharonovich, Z. Wang, X. Yuan, S. Castelletto, S. Prawer, and M. Atatüre, Physical Review B—Condensed Matter and Materials Physics **86**, 195210 (2012).

[5] G. D. Marshall, T. Gaebel, J. C. Matthews, J. Enderlein, J. L. O'Brien, and J. R. Rabeau, New Journal of Physics **13**, 055016 (2011).

[6] H. Utzat, W. Sun, A. E. Kaplan, F. Krieg, M. Ginterseder, B. Spokoyny, N. D. Klein, K. E. Shulenberger, C. F. Perkinson, M. V. Kovalenko, et al., Science **363**, 1068 (2019).

[7] V. Zwiller, T. Aichele, and O. Benson, Physical Review B **69**, 165307 (2004).

[8] A. Reigue, J. Iles-Smith, F. Lux, L. Monniello, M. Bernard, F. Margaillan, A. Lemaitre, A. Martinez, D. P. McCutcheon, J. Mørk, et al., Physical review letters **118**, 233602 (2017).

[9] C. Schimpf, M. Reindl, P. Klenovskỳ, T. Fromherz, S. F. Covre Da Silva, J. Hofer, C. Schneider, S. Höfling, R. Trotta, and A. Rastelli, Optics express **27**, 35290 (2019).

[10] M. von Helversen, L. Greten, I. Limame, C.-W. Shih, P. Schlaugat, C. Anton-Solanas, C. Schneider, B. Rosa, A. Knorr, and S. Reitzenstein, 2D Materials **10**, 045034 (2023).
